# Higher Levels of Care in Young Adult Mental Health

**DOI:** 10.1007/s11920-025-01625-5

**Published:** 2025-08-26

**Authors:** Charissa M. Newkirk, Jennifer J. Cenker, Matthew Phillips, Meera Menon

**Affiliations:** 1 The Ohio State University Wexner Medical Center, 1670 Upham Dr, Columbus, OH 43210 USA; 2 The University of North Carolina at Chapel Hill, 101 Manning Dr, NC, Chapel Hill, NC 27514, USA

**Keywords:** Levels of care, Young adults, Adolescents, Partial hospitalization program, Intensive outpatient program, Psychiatry, Mental health

## Abstract

**Purpose of Review:**

To review recent literature on the key components of partial hospitalization and intensive outpatient programming and their impact on adolescent and young adult patient populations’ mental health.

**Recent Findings:**

There is limited published data examining these levels of care in youth and young adults. Existing data supports use of partial hospitalization and intensive outpatient programming for youth and young adults at high risk of suicide. These programs have been associated with reduced psychiatric-related emergency department visits. Dialectical behavioral therapy (DBT) is a key component across programming given benefits to suicidality. Youth-specific considerations including family therapy, coordination with schools, and unique therapy programming to target substance use or unique challenges faced by high-risk populations.

**Summary:**

Current data supports the use of partial hospitalization and intensive outpatient programming for youth and young adults at high risk of suicide. Additional research examining efficacy of various psychotherapy models as well as more diverse patient populations would help further guide treatment in high-risk patient populations.

## Introduction

Partial Hospitalization Programs (PHP) and Intensive Outpatient Programs (IOP) have long served an important purpose. They provide intermediary mental health care for patients who need more intensive treatment than what the traditional outpatient setting can provide, but do not require the brief, restrictive, and high intensity treatment of an inpatient psychiatric unit. In other words, they can provide a step-up for patients who have acute decompensation of psychiatric symptoms as well as a step-down for those who are being discharged from the hospital but need a more gradual decrease in intensity to maintain safety and build adequate supports [1]. The structure of PHP/IOP programming facilitates the unique purpose of high intensity treatment while still allowing a patient to face stressors, maintain daily functioning, and have necessary social interaction. These types of programs, especially when used in a step-down fashion, have been shown to prevent rehospitalization during the high-risk period in the weeks following an inpatient psychiatric hospitalization [2]. This is especially true for younger patient populations.

The adolescent (12–17 years old) and young adult patient populations (18–25 years of age) are at high risk for the onset of many psychiatric illnesses. By the time children are 18 years old, 49% have a diagnosable mental illness, with this number rising to 63% by the age of 25 [3]. In a 2023 report, the rates of counseling and psychotropic medication utilization even prior to attending college has continued to increase, demonstrating the severity of psychiatric conditions amongst adolescents even prior to university enrollment [4]. Due to the increasing severity of these disorders diagnosed in adolescence and young adulthood, there appears to be an associated rise in psychiatric crises. In this population, psychiatric emergency services utilization tripled from 2007 to 2017, hospitalization rates tripled since 1994, and suicidal ideation and attempts have climbed between 2010 and 2017 [5]. The COVID-19 pandemic especially contributed to the ongoing increased prevalence of psychiatric conditions in young adults, with data reflecting increases in depression, anxiety, inattention, suicidal ideation, suicide attempts, and substance use [6].

Currently, there is an imbalance between the resources available to the young adult population and those needed to adequately meet their needs. While not all young adults attend college, much of the data on this topic does examine the experience of young adults in college. As of 2016, an overwhelming majority of staff at college counseling centers report a substantial increase in students seeking treatment as well as presenting with more severe psychiatric concerns. However, only about 60% of universities have a psychiatrist on staff within their health centers [7]. These statistics have not changed much in recent years. According to the 2024 Association for University and College Counseling Directors Survey, only 64.6% of college campuses have access to psychiatric services. On average, the number of psychiatrist hours accessible to students is 24.3 per week [8]. Students requiring hospitalization are faced with unique challenges. This includes lack of communication between the hospital and the university regarding returning to school, which is perpetuated by siloes within systems and by hospital staff having less knowledge of the unique needs of students. Parents of young adults in college face the added stress of determining whether their child should remain enrolled and managing tuition payments while their child seeks a higher level of care. Additionally, students are often covered under a parent’s health insurance, which may allow access to some services (such as student health) but not others (such as hospitalizations or specialized outpatient care). Insurance coverage may also be limited if the parent’s plan only provides in-network benefits in a region far from where the young adult is living. Furthermore, many students are navigating the healthcare system for the first time independently and may lack the health literacy needed to understand their insurance coverage. These barriers, confusing for both young adults and parents, can lead to delays in accessing appropriate levels of care, leaving patients more vulnerable to psychiatric crises [2].

With these recent findings, there has been a call by many to address the needs of this patient population beyond the current system of care that exists for young adults. While hospitalization can help to stabilize patients with severe mental illness in the short-term, it does not necessarily address ongoing factors that contribute to chronic disease and less so addresses factors specific to the young adult population. Given the current gaps in care within the college mental health system, PHP and IOP programs tailored to the young adult population are beneficial. In this review, we examine currently published data on PHP and IOP programming for both adolescents and young adults. We will discuss the infrastructure of various programs and relevant data regarding treatment outcomes. Our goal is to offer plausible avenues for universities and hospital systems to address a rising need for psychiatric care in adolescents and young adults.

## PHP/IOP Programming in Adolescents

Many outpatient programs serve a mixed population of both adolescents and young adults. To capture the full scope of data on higher levels of care in young adults and to reflect the increasing prevalence of mental illness beginning in adolescence, we found it relevant to include discussion of the adolescent population. Accordingly, we will address both mixed-age programs and those specifically designed for adolescents. We also will include information regarding specifically the LGBTQIA + community as this population is at particularly higher risk for suicidal behaviors and has subsequently been the focus of more recent literature.

Much like the young adult population, the data specifically examining higher levels of care within the adolescent population is limited. However, there have been some relevant studies that have shown promising outcomes for high-risk adolescents. One recent review examined PHP programs in multiple countries. In this review, PHPs were characterized as programs that did not require 24-hour supervision, offered at least 20 h per week of treatment, and took an interdisciplinary approach (i.e. medication management along with multiple therapy modalities including individual, family, and group therapy). In the programs evaluated, there was evidence of symptom improvement upon discharge compared to intake assessments. This trend was true specifically among adolescents with multiple co-morbid conditions [9]. It is especially important to highlight the efficacy of these programs given the high-risk timeframe for adverse outcomes following psychiatric hospitalization in the pediatric population. There is evidence to suggest that prompt follow-up in the outpatient setting within 7 days of discharge alone decreases suicide risk within 6 months following discharge in the adolescent population [10]. Therefore, an argument can be made that avenues to deliver quality intensive outpatient programming are important to pursue.

Prior to the COVID-19 pandemic, there were noted disparities in psychiatric centers that offer these programs. Such limits included geographic location, program criteria that excluded adolescents, and ability to physically be present for programming multiple days a week. These disparities were only heightened by the COVID-19 pandemic, and thus it became important for this type of programming to be restructured to include more remote platforms. Since 2020, there has been more specific literature examining the impact of online programming on patient outcomes. There has been data suggesting that online mixed child and young adult programming has resulted in a significant decrease in psychiatric-related emergency department presentations even up to three months after programming has ended. This was supported across multiple patient demographics including age, gender, race, and sexual orientation [11]. Additional studies using PHQ-9 data have shown that adolescents and young adults had improvement in scores on discharge compared to intake in online IOP programming. This decreased score was maintained even up to 3 months after program cessation, suggesting sustained effects [12].

One component that has been prevalent across programming targeted towards the adolescent population regardless of in-person vs. online format is Dialectical Behavioral Therapy (DBT). DBT has historically been utilized in adults as treatment for a variety of disorders and symptoms, more specifically as evidence-based therapy for Borderline Personality Disorder, suicidality, and self-injurious behaviors. Given its use in adults, DBT has also further been studied in adolescent populations specifically related to suicidal ideation and self-harming behaviors. Randomized control trials have compared DBT with supportive therapy and found DBT to be more efficacious in reducing repeated suicide attempts and self-harming behaviors in high-risk adolescents [13]. DBT has therefore been extended into partial hospitalization programs both in mixed programs with adolescents and young adults as well as adolescent-specific programs. Specifically, DBT has been shown to demonstrate symptoms improvement in suicidal behaviors, depression, and anxiety even within the shorter timeframe that PHP provides as compared to traditional outpatient therapy [3, 9]. Specific versions of DBT, such as Dialectical Behavioral Therapy for Adolescents (DBT-A) have even been developed for adolescents who experience suicidal ideation and self-injury to help promote skills in emotional regulation. DBT-A integrates components of DBT plus additional features such as validating multiple possible perspectives when analyzing scenarios, self-acceptance, and analyzing the adolescent-parent relationship. In randomized controlled trials, it has shown promising results in reducing suicidal ideation and self-injurious behaviors in adolescents [14]. When utilized in a PHP setting, DBT-A proved efficacious in decreasing depressive symptoms [15].

## PHP/IOP Programming in Young Adults

As adolescent patients transition into young adulthood, new and unique challenges appear, especially among those pursue higher levels of education. After high school, these patients enter settings with less structure and support than they may have previously received in a school setting or at home with family. This change in structure is seen both from a psychosocial perspective (e.g., creating a daily routine, navigating peer social groups) as well as from a healthcare perspective (e.g., “aging out” of pediatric healthcare, out of state students choosing between care at home or at their university, lack of mental health resources on campuses, acquiring healthcare literacy). This is further compounded by the fact that many psychiatric disorders, from mood disorders to psychotic disorders, often emerge during this developmental period. Currently, there is a lack of robust data regarding outcomes for young-adult specific PHP and IOP programs; many programs take a mixed population approach. However, we will review current models that exist for the young adult patient as potential pathways for broader implementation that appear to have been successful for patients and may address some of the gaps in care that exist for higher risk patients.

PHP and IOP programs created for young adults are subjectively preferred by patients within these programs as compared to general adult PHPs and IOPs. In general adult PHPs and IOPs, retention rates of young adults tend to be lower. This is likely because group settings can be difficult for young adults due to differences in life stages among peers, concern for significant academic disruptions if the young adult is enrolled in college, or concern for significant work disruption if the young adult is newly establishing themselves in their career. However, upon introduction of young adult specific programming, patients can have more profound group discussions due to neurodevelopmental similarities (e.g., identity formation, development of personal values). This subsequently leads to more social connectedness amongst those in the group together and thus serves as a motivator for program retention. This, along with offering accessible times for students (i.e., not early morning or late evening), has appeared to be correlated with higher retention rates in this age-specific group compared to general adult groups [16].

To address concerns for students having to decide to seek higher level treatment at home versus closer to school, some institutions have begun to have intensive programming on college campuses. DBT continues to be a highlight for these programs given its known effects on suicidal ideation and behaviors. These programs also offer CBT, allowing for other important skills to be taught such as recognizing maladaptive thought patterns and understanding of emotions [16]. As with adolescent populations and other mixed population IOPs, there have been formations of DBT specific to IOP programs targeting high risk college students given the broader data DBT has for depression and anxiety symptoms, impulsivity, handling intense emotions, and managing interpersonal conflict [5].

Disruptions in care that occur during the transition from pediatric to adult care are associated with higher risk for suicide [17]. Conversely, timely psychiatric follow up after discharge from psychiatric hospitalization is associated with reduced risk of suicide [1]. Thus, an essential component of programs designed for young adults is the coordination of care. This involves ensuring that staff are aware of disability accommodations available to university students, understanding the varying levels of parental involvement on a case-by-case basis, and determining whether patients are better suited for on-campus services or community-based programs, depending on what is already offered by the university. This process requires ongoing communication with university personnel to effectively coordinate individual care. Additionally, from a systems-level perspective, it is crucial to gather feedback to ensure that these programs continue to meet the evolving needs of the student population [17]. However, even for young adults who are not pursuing higher levels of education, there are still elements that can be incorporated to help target challenges in the workforce. One example is incorporation of services to help with finding and maintaining employment as well as maintaining daily structure [16].

## Special Considerations for LGBTQIA+ Youth and Young Adults

Within the adolescent and young adult populations, a subgroup that consistently has been flagged as high risk for suicide is LGBTQIA + community. Even at younger ages, this population tends to experience social exclusion and victimization compared to LGBTQIA + adults. Over time, this population tends to experience chronic stressors related to stigmatization (e.g., lack of support at school, familial conflict, etc.) and thus puts these patients at higher risk of developing a psychiatric disorder in their adolescent and young adulthood years. These include diagnoses such as mood disorders, anxiety disorders, and PTSD and symptoms including suicidal ideation [18]. When placed in the context of higher levels of care, LGBTQIA + patients tend to have more severe presentations of depression, self-injurious behavior, and suicidal ideation on intake into these programs. Evidence examining college students, including undergraduate, graduate, and professional levels, has shown LGBTQIA + students tend to be at higher risk of psychiatric hospitalization for suicidality [19]. The psychiatric risk for this population has only heightened since the COVID-19 pandemic as LGBTQIA + patient tend to hold jobs in industries affected by the pandemic (such as food service) or are in academic settings that were disrupted by the pandemic. These stressors, along with financial strain and insurance barriers, have likely contributed to this population’s continued higher risk for psychiatric decompensation since 2020 [20]. However, there have been reports of programming tailored towards this patient population with promising results. In one study that examined a mixed (adolescent and young adult) remote intensive outpatient program with programming specific to the LGBTQIA + population, there was a significant reduction in depressive symptoms and suicidal ideation at the time of discharge from the program [21]. This suggests that even amongst severely high risk and vulnerable populations, intensive outpatient programming can be extremely beneficial.

## Special Considerations for Patients Struggling with Substance Use

An additional challenge that is relevant to the young adult population is alcohol and substance use. This is especially true for patients who already have underlying psychiatric disorders. Among college students receiving mental health care, 40% report binge drinking and 24% report using marijuana within the last two weeks [17]. Since the COVID-19 pandemic, 25% of young adults have reported increased nicotine use (including use of e-cigarettes and vapes). Additionally, there have been several studies globally that reported increased alcohol consumption during the COVID-19 pandemic [6]. However, substance use treatment is commonly integrated into IOPs specific to young adults. At a program for 18–24-year-olds in Pittsburgh, Pennsylvania, harm reduction strategies and psychoeducation on how substance use contributes to psychiatric symptoms were incorporated into the curriculum. Additionally, psychoeducation was given regarding interactions between substances and psychiatric medications [17]. However, the cross section between substance use, psychiatric disorders, and increased rates of utilization of higher levels of care is an area for further research.

**Table 1:** Unique aspects of IOPs and PHPs in youth and young adult patients

Special considerations	-Increased emphasis on family structure and family therapy -Increased need for coordination with schools/educational structures -Flexible timing needed to allow for continued education and reduce attrition rates -Interfaces with youth during high-risk period for development of mood and psychotic disorders
Common Aspects of Programs	-DBT plus additional features to target acceptance -CBT to recognize maladaptive thought patterns -Programming specific to target substance use and support high risk patient populations -Common themes include identity and value formation

## Conclusions

Recent literature supports the use of partial hospitalization and intensive outpatient programming to target suicidal ideation in adolescent and young adult populations. Common features of programming and unique considerations for youth that have been explored throughout this review are summarized in Table 1. Despite generally limited studies available for review, these programs have been shown to lead to significantly reduced emergency department admissions from intake to 3 month follow up [11]. LGBTQIA + patients, who are at particularly high risk for suicide, have demonstrated promising improvements to depressive symptoms at time of discharge [21]. PHP and IOP may help meet the increasing needs for mental health services that has led to increased boarding times among youth since the COVID-19 pandemic [22]. Future considerations for study include further examining the degree of persistence of treatment gains after programming, as most studies focused on immediate or 3 month follow up after discharge from program. Further studies would also benefit from examining more diverse populations, as current studies tended to look at white patients potentially due to the economics of these programs which tend to take private insurance or require initial out-of-pocket payment and wait for reimbursement [9]. In addition, further studies examining specific components of programming, i.e., comparing CBT versus DBT models, would help guide program development.

## Data Availability

No datasets were generated or analysed during the current study.
